# Characteristics of the Fecal Microbiome of Piglets with Diarrhea Identified Using Shotgun Metagenomics Sequencing

**DOI:** 10.3390/ani13142303

**Published:** 2023-07-14

**Authors:** Mariya Gryaznova, Yuliya Smirnova, Inna Burakova, Polina Morozova, Ekaterina Nesterova, Mariya Gladkikh, Evgeny Mikhaylov, Mikhail Syromyatnikov

**Affiliations:** 1 Laboratory of Metagenomics and Food Biotechnology, Voronezh State University of Engineering Technologies, 394036 Voronezh, Russia; mariya-vg@mail.ru (M.G.); dyd16@mail.ru (Y.S.); vitkalovai@inbox.ru (I.B.); ms.cloud00.00@mail.ru (P.M.); katya.nesterova.1997@mail.ru (E.N.); mariya221095@yandex.ru (M.G.); 2 Department of Genetics, Cytology and Bioengineering, Voronezh State University, 394018 Voronezh, Russia; 3 FSBSI All-Russian Veterinary Research Institute of Pathology, Pharmacology and Therapy, 394061 Voronezh, Russia; voronezh81@rambler.ru

**Keywords:** fecal microbiome, shotgun metagenomics sequencing, diarrhea, piglets

## Abstract

**Simple Summary:**

Diarrhea in newborn piglets is one of the important problems for the agricultural industry. It is believed that this disease is associated with changes in the intestinal microbiome. The present work is aimed at studying the composition of the microbiome of newborn piglets with diarrhea in order to identify relevant markers of the disease. Our comprehensive microbiome study did not reveal bacterial and eukaryotic aberrations in the fecal microbiome of diarrheal piglets; at the same time, we observed higher levels of bacterial diversity, which may be associated with dysbacteriosis and inflammation. In the observation group, an increase in the abundance of *Bacteroides* B40-8 phage was also recorded, and in the healthy group, an increase in the abundance of *Escherichia* virus BP4. Thus, the results of our study show the ambiguity of the previously proposed relationship between the bacterial community of the fecal microbiome of suckling piglets and the development of diarrhea, and also indicate the need for further research in this area.

**Abstract:**

Diarrhea in piglets is one of the most common diseases leading to high mortality and, as a result, to economic losses. Shotgun metagenomic sequencing was performed on the DNBSEQ-G50, MGI system to study the role of the fecal microbiome in the development of diarrhea in newborn piglets. Analysis of the study data showed that the composition of the fecal microbiome at the level of bacteria and fungi did not differ in piglets with diarrhea from the healthy group. Bacteria belonging to the phyla *Firmicutes*, *Bacteroidetes*, *Proteobacteria*, *Actinobacteria*, and *Fusobacteria* were the most abundant. However, a higher level of bacterial alpha diversity was observed in the group of piglets with diarrhea, which may be due to dysbacteriosis and inflammation. The study of the virome showed the difference between the two types of phages: *Bacteroides* B40-8 prevailed in diseased piglets, while *Escherichia* virus BP4 was found in greater numbers in healthy piglets. The results of our study suggest that the association between the fecal microbiome and susceptibility to diarrhea in suckling piglets may have been previously overestimated.

## 1. Introduction

In recent years, there has been an increase in the production of animal products for human consumption around the world. Like most farm animals raised for food, pigs and piglets suffer from various diseases [1,2], one of which is diarrhea in piglets. Economic losses associated with increased morbidity and mortality affect the livestock industry [3,4].

Like all animals, pigs have a complex intestinal community that includes bacteria, viruses, archaea, and fungi. The development of the microbiome of the mammalian intestinal tract begins at birth and continues to dynamically develop as the animal matures under the influence of a large number of factors [5,6].

Importantly, the fungal composition of the fecal microbiome in newborn piglets is less understood than bacteria, and more research is needed to fully understand the diversity and function of fungi in the piglet fecal microbiome. However, some fungi present in the fecal microbiome of healthy newborn piglets can influence immune responses as well as alter the severity of certain diseases [7,8,9,10]. In addition, species have been found to have probiotic properties as well as produce beneficial metabolites and enzymes that play a role in gut health [10,11,12].

The study of viruses and bacteriophages in the fecal microbiome of newborn piglets is an emerging area of research, and our understanding of their presence and role is still evolving. It should be noted that some viruses and bacteriophages found in the intestinal microbiome of newborn piglets infect the gastrointestinal tract and may play a role in the dynamics of the intestinal microbiome [13,14].

The totality and interaction of these biomes make a fundamental contribution to the metabolism, physiological processes, immunity, and health of the animal [15,16,17]. Based on these data, the study aimed to determine the complete composition of the fecal microbiome and identify species associated with the development of diarrhea in newborn piglets. To achieve this goal, whole genome sequencing was performed on the DNB-SEQ-G50 system (MGI, Shenzhen, China). This platform has several advantages over other sequencing methods, such as high performance; DNBSEQ-G50 can generate up to 1.2 terabases of data per cycle, making it suitable for large-scale genomic research. In addition, this system is highly accurate, using combinatorial probe–anchor synthesis (CPAS) technology, which provides an accurate basic call with a low error rate [18]. The DNBSEQ-G50 can be used for a wide range of sequencing applications, from targeted resequencing to whole genome sequencing and transcriptomics. It is also easy to use with its user-friendly interface, sample preparation that requires small amounts of starting DNA or RNA, and the sequencing process itself. In addition, DNBSEQ-G50 can generate high-quality sequencing data in a relatively short time, allowing researchers to obtain results faster than with some other platforms [19,20].

## 2. Materials and Methods

### 2.1. Experimental Design and Collection of Faecal Samples

A total of 16 piglets of 1–5 days of age (F1 hybrids) were obtained by crossing animals of the Large White and Landrace breeds. Animals were divided into two equal groups. The control group included 8 healthy piglets, and the observation group included 8 piglets with severe diarrhea observed from the 2nd day of life. The animals were kept indoors at a temperature of 30 ± 2 °C and a humidity of 55 ± 7%. The diagnosis of diarrhea was made based on the following clinical picture: shaky, unsteady gait because of general dehydration, loose stools, a fetid odor of feces, and general contamination of piglets with feces. Microscopy of feces did not reveal any pathogens of parasitic diseases. Piglets received mother’s milk from sows. The average weight of piglets in the healthy group was 1.5 ± 0.45 kg, and in the sick group was 1.1 ± 0.38 kg. For the study, stool samples were taken in the amount of 10 ± 3 g, placed in microcentrifuge tubes, and delivered to the laboratory on ice.

### 2.2. Microbial DNA Extraction and Shotgun Metagenomics Sequencing

The commercial HiPure DNA Micro Kit (Magen, Guangzhou, China) was used for DNA extraction from the obtained samples. Isolation was carried out according to the manufacturer’s protocol. Libraries were prepared according to the following protocol: DNA was fragmented using the MGIEasy Fast FS Library Prep Module kit (MGI, Shenzhen, China), followed by purification with MGIEasy DNA Clean Beads (MGI, Shenzhen, China) magnetic particles, ligation of the adapters with the MGIEasy UDB Primers Adapter Kit A (MGI, Shenzhen, China), and PCR amplification. The quality of the DNA library was assessed using the Qubit bioanalyzer and the Qubit dsDNA HS Assay Kit (Invitrogen, Waltham, MA, USA).

Further circularization of one strand was performed using the MGIEasy Dual Barcode Circularization Module (MGI, Shenzhen, China). The final libraries were pooled and sequenced using the MGI DNBSEQ-G50 sequencing platform with DNBSEQ-G50RS Sequencing Flow Cell Model: FCL (MGI, Shenzhen, China). DNBSEQ-G50RS High-throughput Sequencing Kit Model: FCL PE100 (MGI, Shenzhen, China) was used to create DNB.

### 2.3. Bioinformatics and Statistical Analysis

Raw metagenomic data were quality assessed using FastQC [21]. Technical sequences and low-quality bases (Q < 30) were trimmed with the fastp [22]. The human and host sequences from samples were removed by mapping metagenomic reads to hg38 human and domestic pig (GCA_017957985.1) reference genomes using the Bowtie2 tool [23]. Taxonomic profiling of samples was performed using Kraken2 with standard bacterial, viral, and eukaryotic databases [24]. The Pavian tool was used for the interactive visualization of the Kraken2 output [25]. Resistance antibiotics gene profiles were obtained using GROOT with ARG-ANNOT precomputed index [26,27].

Alpha-diversity calculation was performed using the MicrobiotaProcess package [28]. The difference in alpha diversity was estimated using the Wilcoxon rank sum test [29]. Bidimensional visualization was performed using non-metric multidimensional scaling and the Bray–Curtis dissimilarity metric implemented in MicrobiotaProcess packages for GNU/R [28]. The ADONIS function was used for obtaining differences in the taxonomic compositions of the observation group. An analysis of species differential abundance was performed using the metagenomeSeq package [30]. An adjusted *p*-value ≤ 0.05 was considered statistically significant.

## 3. Results

**Figure 1:**
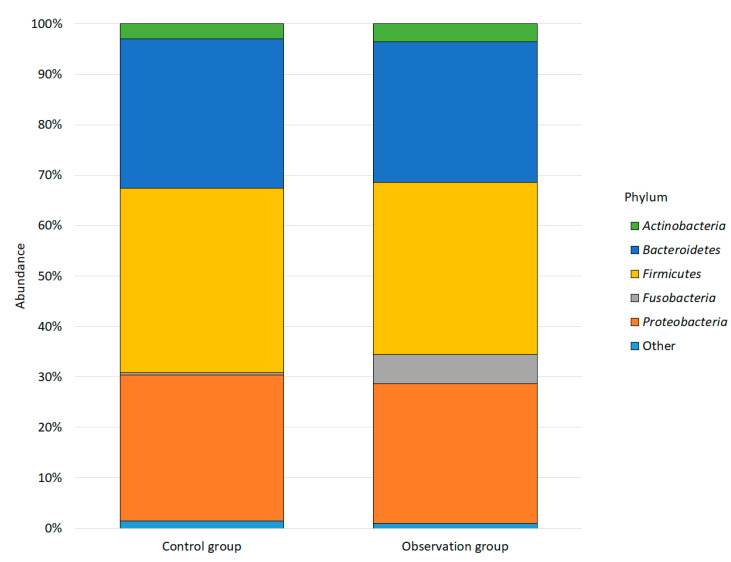
Relative abundance of bacterial phyla observed in the groups. Only top taxa are reported.

In this study, we analyzed the microbiota as well as viral and eukaryotic profiles in the feces of 8 healthy piglets and 8 piglets with diarrhea using shotgun metagenomic sequencing in the DNBSEQ-G50 system (MGI, Shenzhen, China). As a result of sequencing, we received about 109.5 million pairs of reads. After fine pruning and deletion of host and human sequences, approximately 28.6 million reads remained (an average of 1.8 million read pairs per sample). Read-based shotgun sequence classification produced using the standard Kraken2 database identified 7718 bacterial species. Among the identified genomes, bacteria of 41 phyla were found, among which representatives of the phyla *Firmicutes* predominated in both studied groups (36.52% in the control group and 34.15% in the observation group), *Bacteroidetes* (29.62% in the control group and 27.95% in the observation group), *Proteobacteria* (29.01% in the control group and 27.68% in the observation group), *Actinobacteria* (2.98% in the control group and 3.52% in the observation group), and *Fusobacteria* (0.46% in the control group and 5.73% in the observation group); the phyla with an average abundance of less than 1.0% for one of the studied groups were combined into “Other” (Figure 1).

**Figure 2:**
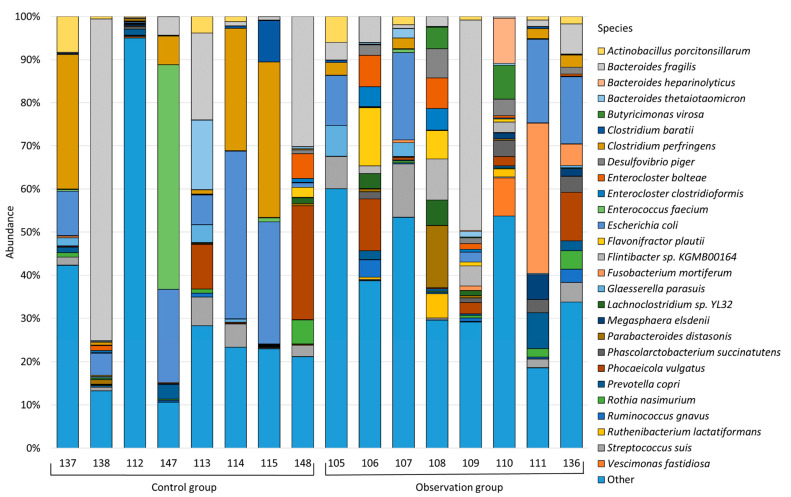
Bacterial composition of the studied samples at the species level.

Species with an average abundance for one of the studied groups of less than 1.0% were combined into “Other”; thus, Figure 2 shows the top bacterial species for each sample. See Table S1 for the complete bacterial composition of test samples.

Upon closer examination, it was found that the *Bacteroides fragilis* species prevailed in the healthy group of piglets and the group of piglets with diarrhea, and its abundance was 16.43% and 8.84%, respectively. The next largest for both groups was the genus *Escherichia coli* (14.03% in the healthy group and 8.68% in the observation group). In the healthy group, the next largest were *Clostridium perfringens* (13.07%), *Enterococcus faecium* (6.71%), *Phocaeicola vulgatus* (4.70%), *Streptococcus suis* (2.23%), *Bacteroides thetaiotaomicron* (2.13%), *Actinobacillus porcitonsillarum* (1.78%), *Clostridium baratii* (1.33%), and *Rothia nasimurium* (1.02%). In the group of piglets with diarrhea, after *Bacteroides fragilis* and *Escherichia coli*, we observed the following population distribution: *Fusobacterium mortiferum* (5.19%), *Phocaeicola vulgatus* (3.61%), *Streptococcus suis* (3.43%), *Flavonifractor plautii* (2.72%), *Flintibacter* sp. KGMB00164 (2.29%), *Enterocloster bolteae* (2.09%), *Desulfovibrio piger* (2.02%), *Parabacteroides distasonis* (2.01%), *Prevotella copri* (1.80%), *Phascolarctobacterium succinatutens* (1.68%), *Butyricimonas virosa* (1.63%), *Actinobacillus porcitonsillarum* (1.42%), *Glaesserella parasuis* (1.41%), *Bacteroides heparinolyticus* (1.38%), *Lachnoclostridium* sp. YL32 (1.36%), *Clostridium perfringens* (1.33%), *Enterocloster clostridioformis* (1.33%), *Vescimonas fastidiosa* (1.13%), *Megasphaera elsdenii* (1.11%), *Ruminococcus gnavus* (1.11%), and *Ruthenibacterium lactatiformans* (1.01%). The abundance of other genera was less than 1%.

Despite these contrasts in microbiome composition, differential abundance analysis did not reveal statistically significant differences between the studied groups.

**Figure 3:**
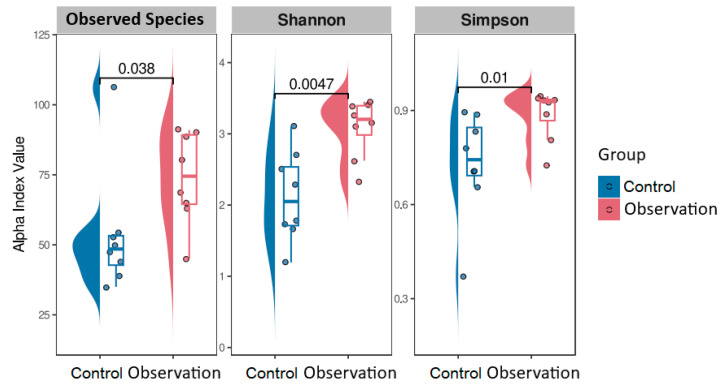
Microbiome alpha-diversity of study groups with *p*-values.

We also evaluated alpha and beta diversity in the fecal microbiota of piglets. Estimating the microbial diversity of feces using our shotgun dataset, we obtained lower alpha diversity in the healthy group compared to the observation group (Figure 3).

**Figure 4:**
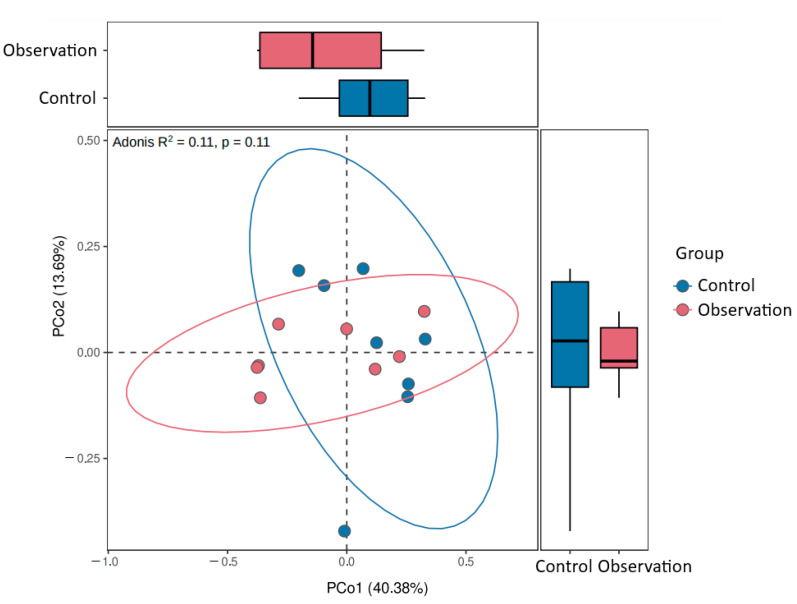
Principal coordinate analysis (PCoA) plot of the beta-diversity based on the Bray–Curtis dissimilarity index between microbial community samples in the study groups.

Distance analysis of beta diversity did not reveal group-specific clustering (Figure 4).

In addition to the bacterial composition, the eukaryotic and viral composition of the samples were also studied during the investigation.

In the test samples, 80 species of fungi were identified (see Table S2), among which the *Schizosaccharomyces pombe* species prevailed in both groups (8.59% in the healthy group and 10.81% in the observation group). An analysis of alpha and beta diversity and a differential analysis of the abundance of the eukaryotic composition did not show statistically significant differences.

**Figure 5:**
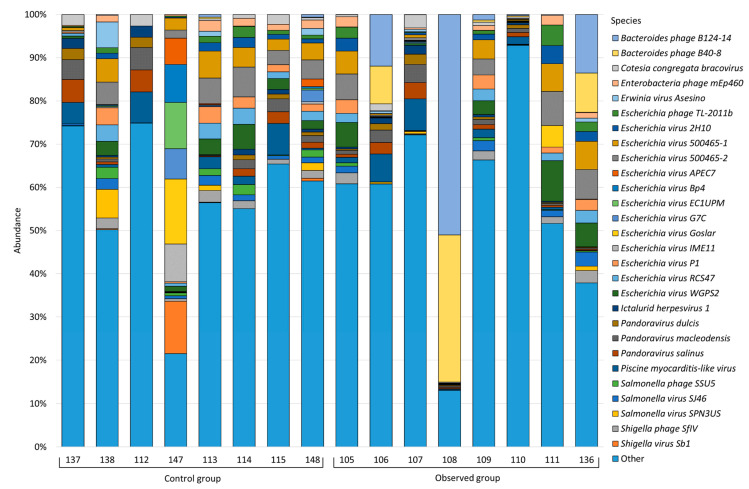
Viral composition of the studied samples.

Classification of viruses, as well as bacteria and fungi, was performed using Kraken2, which identified 4040 virus species that are part of 144 families (see Table S3 for a complete list). Figure 5 shows viruses greater than 1.0%, species below this threshold are grouped under “Other”.

In the healthy group, we observed the following virus distribution: *Escherichia* virus 500465-2 (3.53%) was characterized by the highest relative abundance, then *Escherichia* virus 500465-1 (3.28%), *Piscine myocarditis-like* virus (3.17%), *Escherichia* virus WGPS2 (2.37%), *Pandoravirus macleodensis* (2.17%), *Pandoravirus salinus* (2.12%), *Escherichia* virus RCS47 (2.04%), *Escherichia* virus Goslar (1.90%), *Escherichia* virus P1 (1.79%), *Shigella* virus Sb1 (1.65%), *Escherichia* virus EC1UPM (1.44%), *Shigella* phage SfIV (1.32%), *Escherichia* virus G7C (1.28%), *Salmonella* virus SPN3US (1.22%), *Escherichia* virus IME11 (1.21%), *Enterobacteria* phage mEp460 (1.21%), *Cotesia congregata* bracovirus (1.20%), *Escherichia* virus Bp4 (1.19%), *Salmonella* virus SJ46 (1.18%), *Salmonella* phage SSU5 (1.13%), *Erwinia* virus *Asesino* (1.11%), *Pandoravirus dulcis* (1.09%), *Ictalurid herpesvirus* 1 (1.06%), *Escherichia* virus APEC7 (1.03%), and *Escherichia* virus 2H10 (1.00%). The abundance of other viral species was less than 1.00%.

In the group of piglets with diarrhea, *Bacteroides* phage B124-14 prevailed with the highest relative abundance (9.78%); then, we observed the following distribution: *Bacteroides* phage B40-8 (6.58%), *Escherichia* virus 500465-2 (3.19%), *Escherichia* virus WGPS2 (3.07%), *Escherichia* virus 500465-1 (2.99%), *Piscine myocarditis-like* virus (2.45%), *Escherichia* virus 2H10 (1.42%), *Pandoravirus macleodensis* (1.39%), *Escherichia* phage TL-2011b (1.33%), *Escherichia* virus P1 (1.32%), *Pandoravirus salinus* (1.28%), *Escherichia* virus RCS47 (1.26%), *Shigella* phage SfIV (1.18%), and *Salmonella* virus SJ46 (1.12%).

**Figure 6:**
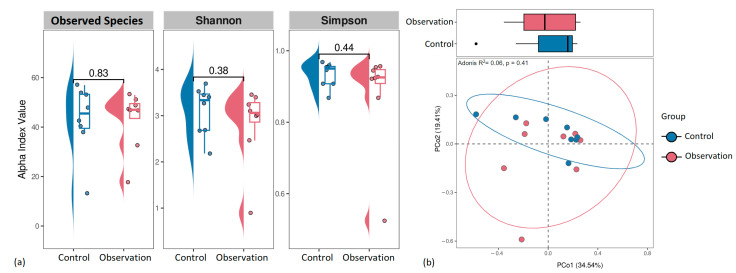
Virus alpha and beta diversity measures of study groups with *p*-values. (**a**) Alpha diversity parameters show no difference between groups. (**b**) Principal coordinate analysis (PCoA) plot of the beta diversity based on the Bray–Curtis dissimilarity index between viral communities.

Despite the presence of differences in the distribution of viruses between the studied groups, we did not find statistically significant differences in alpha and beta diversity (Figure 6).

An analysis to examine differences in abundance at the species level revealed that the prevalence of the two viruses had a statistically significant difference between groups. Thus, the abundance of *Bacteroides* phage B40-8 was higher in the group of piglets with diarrhea and amounted to 6.58%, compared with the healthy group (0.06%, *p*-value = 0.04). The abundance of *Escherichia* virus Bp4 in the healthy group was 1.19%, and 0.002% in the observation group (*p*-value = 0.04).

## 4. Discussion

Our study aimed to identify differences in the microbiome between suckling piglets with diarrhea and a group of healthy ones. The study of the microbiome profile was carried out using the DNBSEQ-G50 sequencing system. Jaehoon Jung and colleagues showed how synbiotics affect the fecal microbiome of Korean black pigs. For the study, they used shotgun metagenome sequencing in the MGISEQ system, as well as sequencing of the target *16S rRNA* gene. They found statistically significant effects of synbiotic treatment on the distribution of microbial functional gene groups using shotgun sequence data, but not with *16S rRNA* sequence data [31].

The types of bacteria actively colonizing the intestines of newborn piglets are gram-positive *Firmicutes* and *Actinobacteria*, as well as gram-negative *Fusobacteria* and *Bacteroidetes* [32,33,34]. The most common type in our study was *Firmicutes*. The maximum abundance of *Proteobacteria* is characteristic of newborn piglets. Our study also showed a significant predominance of *Fusobacteria* in the observation group compared to the control group, but this difference was not statistically significant. Despite this, it should be noted that there are more *Fusobacteria* in suckling piglets than in piglets at other growth stages. *Fusobacteria* and other pathogenic bacteria commonly found in the gastrointestinal tract of suckling pigs are known to cause diseases such as diarrhea and enteritis [35,36,37]. In addition, it has been observed that the number of *Fusobacteria* in piglets with diarrhea can be four times higher than in healthy piglets. Such disturbances not only increase the risk of diarrhea and mortality but can also affect nutrient absorption and host anti-inflammatory regulation [38].

It has been noted that suckling pigs are characterized by the predominance of *Bacteroidaceae* over *Prevotellaceae* [12]. We observed this pattern in our study for both groups. Healthy piglets had more *Enterococcus faecium* than piglets with diarrhea. It is known that some strains of *E. faecium* are probiotics and can have a beneficial effect on the host organism, reducing the likelihood of developing intestinal pathologies, and strengthening the intestinal wall [39,40,41].

In our study, the number of *Bacteroides fragilis* in the healthy group was even twice as high as in piglets with diarrhea. *B. fragilis* is known to be a symbiont that colonizes the mucus or colonic epithelium. It is known that it can protect the host against multiple preclinical colitis and other gastrointestinal diseases by inducing an anti-inflammatory response [15,42].

We also observed a high abundance of *Clostridium perfringens* in healthy piglets compared to piglets with diarrhea. However, *C. perfringens* is a common pathogen in animals and humans, and some strains are capable of causing diarrhea in newborn piglets [43,44].

However, in all the differences in the bacterial composition noted by us, they were not statistically significant, which may be due to the main limitation of this study—the small sample size.

During the observation, we were interested not only in the bacterial composition of the intestine but also in fungal and viral ones. We found that all fungal species identified belonged to three phyla, the most common of which was *Ascomycota*. *Basidiomycota* was the next most common fungal phylum. Our results are consistent with the literature data [11,45]. *Microsporidia* were another type discovered, with an average abundance for both groups of less than 0.5%. Microsporidia species can cause diarrhea, malabsorption, and possible lung pathology, and host health is a major factor influencing the likelihood of developing these symptoms [46,47]. The most well-known pathogenic species is *Enterocytozoon bieneusi*, which was not found in this study; however, we found *Encephalitozoon romalae, Encephalitozoon enteralis, Encephalitozoon cuniculi,* and *Encephalitozoon hellem*, which can also adversely affect the host [48,49].

The most common fungal genus in the study population was *Aspergillus oryzae*. *A. oryzae* is a fungus commonly used in the fermentation of feed ingredients in pig production. When used in appropriate amounts, *A. oryzae* is generally considered safe and may even provide some health benefits to pigs, such as improved digestibility of feed ingredients and enhanced immune function [50,51]. The genus *Pyricularia* was the second largest among the studied piglets. Representatives of this genus are known to be pathogens for some plants, such as rice and other cereals, and cause blasts in them [52,53]. In our samples, *Pyricularia grisea* accounted for the largest share.

*Schizosaccharomyces pombe* was also found in the studied samples. However, information on the health effects of *S. pombe* in pigs is limited. In turn, supplementation with *S. pombe* has been shown to improve nutrient uptake and growth performance in broiler chickens [54].

We also found that *Fusarium* abundance in diarrheic piglets was not significantly higher than in healthy piglets. *Fusarium* species are known to frequently contaminate grain and other feed ingredients and can produce a variety of mycotoxins including deoxynivalenol, zearalenone, and fumonisins. These mycotoxins can cause negative health effects in pigs [55,56].

*Pichia* and *Penicillium* abundances did not differ between study groups, indicating that the two opportunistic pathogens were not the cause of diarrhea in weaned piglets. Overall, mycobiota analysis did not reveal significant differences in fungal composition between healthy piglets and piglets with diarrhea.

Moreover, part of our work was the study of the intestinal virome of piglets. According to the results, *Siphoviridae* is one of the dominant families both in the observation group and in the healthy group. According to previous studies, *Siphoviridae* accounted for the largest proportion of the porcine intestinal virome and also represented the majority of viruses in the human intestine [44,57]. The other dominant family was the *Myoviridae*, whose abundance between healthy and diseased piglets was almost the same. These data are consistent with previous studies, where this family was one of the most common in pigs [58]. The number of *Schitoviridae* in the group of healthy piglets was significantly higher than in sick ones; however, we were unable to find data on the association of this virus family with the intestines of pigs. It is only known that *Schitoviridae* were previously found in the intestines of horses and wild pandas, as well as in the oral cavity and saliva of humans [59,60,61]. The number of *Podoviridae* was twice as high in piglets with diarrhea. The *Podoviridae* are known to contain viruses that most commonly infect bacteria and dominate all microbiomes. It was previously detected in pig feces [62]. Although these differences are not statistically significant, these results, in combination with previous studies, may indicate that these families may be involved in the pathogenesis of metabolic disorders in the body.

When examining the virome of piglets, we found statistically significant differences between the two bacteriophages. Thus, the number of *Bacteroides* phage B40-8 was higher in the group of piglets with diarrhea compared to the healthy group. It is known that this bacteriophage can infect several strains of *Bacteroides fragilis*, which, in turn, may be involved in the development of diarrhea in pigs [63,64]. However, in our study, *Bacteroides fragilis* was not involved in the development of diarrhea in piglets.

The amount of *Escherichia* virus BP4, on the contrary, prevailed in the group of healthy piglets compared to diarrheal piglets. BP4 is considered a “lytic” phage, which means that it can cause rapid destruction of *E. coli* cells upon infection by lysis or rupture of the bacterial cell [65]. In our study, the group of healthy piglets was dominated by *E. coli*.

Thus, this and other studies indicate that the porcine fecal virome is a complex and diverse ecosystem that plays an important role in pig health and disease. In turn, further research is needed to fully understand the functions and interactions of the various viruses present in the pig intestine. It is also necessary to evaluate the effect of feed on the composition of the fecal virome of piglets, because it is known that feed can have a significant impact on the fecal microbiome of pigs [66].

## 5. Conclusions

This study aimed to comprehensively explore the microbiome of diarrheic piglets using shotgun metagenomics sequencing on the DNBSEQ-G50 system. We conducted a comparative analysis of the microbial, fungal, and viral composition between healthy suckling piglets and piglets with diarrhea. Such patterns may be useful as early biomarkers of diarrhea susceptibility, as well as lay the foundation for early preventive measures. However, in our study, the fecal microbiota at the level of bacteria and fungi did not differ from the healthy group in piglets with diarrhea. We only observed a higher rate of bacterial alpha diversity in the group of diarrheic piglets, which may be due to dysbacteriosis and inflammation. When studying the virome, we found a difference at the level of two phage species: *Bacteroides* phage B40-8 and *Escherichia* virus BP4, the first of which prevailed in sick piglets, and the second in healthy ones. Further research is needed to evaluate the impact of these phages on gut health.

In addition, our work demonstrates the diversity and abundance of the mycobiome in the feces of piglets. Although they are outnumbered by bacteria, the potential role of fungi in piglet health remains unknown, and future studies are needed to evaluate the health impact of fungal populations. It is also necessary to elucidate the mechanism of fungal colonization of the intestines and feces of piglets.

The results of our study may indicate that the association between the fecal microbiome and susceptibility to diarrhea in suckling piglets may have previously been overestimated. However, this is one of the few studies that shed light not only on the bacterial composition but also studied in detail both the fungal and viral composition of the fecal of suckling piglets.

## Data Availability

Sequencing data are available in the NCBI BioProject database (BioProjectID: PRJNA979345).

## Supplementary Materials

The following supporting information can be downloaded at: https://www.mdpi.com/article/10.3390/ani13142303/s1, Table S1: Complete composition of bacteria; Table S2: Complete composition of fungi; Table S3: Complete composition of viruses.
